# Is There Still Any Role for Oxidative Stress in Mitochondrial DNA-Dependent Aging?

**DOI:** 10.3390/genes9040175

**Published:** 2018-03-21

**Authors:** Gábor Zsurka, Viktoriya Peeva, Alexander Kotlyar, Wolfram S. Kunz

**Affiliations:** 1 Institute of Experimental Epileptology and Neurocognition, University Bonn Medical Center, 53105 Bonn, Germany; gabor.zsurka@ukbonn.de (G.Z.); viktoriya.peeva@ukbonn.de (V.P.); 2 Department of Epileptology, University Bonn Medical Center, 53105 Bonn, Germany; 3 Department of Biochemistry & Molecular Biology, Faculty of Life Sciences, Tel Aviv University, Tel Aviv 69978, Israel; s2shak@tau.ac.il

**Keywords:** mitochondrial DNA, aging, reactive oxygen species, oxidative stress

## Abstract

Recent deep sequencing data has provided compelling evidence that the spectrum of somatic point mutations in mitochondrial DNA (mtDNA) in aging tissues lacks G > T transversion mutations. This fact cannot, however, be used as an argument for the missing contribution of reactive oxygen species (ROS) to mitochondria-related aging because it is probably caused by the nucleotide selectivity of mitochondrial DNA polymerase γ (POLG). In contrast to point mutations, the age-dependent accumulation of mitochondrial DNA deletions is, in light of recent experimental data, still explainable by the segregation of mutant molecules generated by the direct mutagenic effects of ROS (in particular, of HO· radicals formed from H_2_O_2_ by a Fenton reaction). The source of ROS remains controversial, because the mitochondrial contribution to tissue ROS production is probably lower than previously thought. Importantly, in the discussion about the potential role of oxidative stress in mitochondria-dependent aging, ROS generated by inflammation-linked processes and the distribution of free iron also require careful consideration.

## 1. Introduction: The Mitochondrial Theory of Aging and Recent Controversial Findings

The central principles of the mitochondrial theory of aging are that (i) mitochondrially produced reactive oxygen species (ROS) can damage mitochondrial DNA (mtDNA), and (ii) ROS-induced lesions in mtDNA can lead to somatic mutations that accumulate, affect the integrity of respiratory chain, and cause mitochondria-dependent aging [1]. More recent data seem to indicate that mtDNA might be more resistant to oxidative damage than previously thought. Instead, many have suggested that the origin of somatic mtDNA mutations is associated with the fidelity of the mtDNA polymerase γ (POLG) [2,3]. Additionally, there seems to be little experimental support for the *vicious cycle* theory, which attempts to explain the age-dependent accumulation of mutations by proposing a mutation-dependent increase of mitochondrial ROS production that, in turn, would result in elevated oxidative mtDNA damage [1,4]. Rather, the age-dependent increase in the somatic mutation load of mtDNA reported by many groups [5,6,7] can be explained sufficiently by the replicative segregation of mitochondrial mutations [8]. This theory has been supported by evidence that individual cells of aged persons accumulate high levels of only one specific mutation [9,10]. Additionally, the effect of mtDNA mutations on mitochondrial ROS production has been reported to be strongly mutation dependent. Only certain mutations that affect the activity of Complex I and Complex V have been convincingly shown to increase mitochondrial ROS production [11,12], while random mtDNA point mutations do not seem to be associated with elevated oxidative stress [13,14,15].

One of the most important issues relating to the mitochondrial theory of aging is the very low frequency of somatic mutations detected in the mtDNA in tissue samples from older individuals. Obviously, the mitochondrial genome is present in multiple copies (approximately 10 copies per mitochondrium), and it is a well-established fact that intact mtDNA can complement for mutated genomes. Therefore, it is difficult to imagine how minor changes in the mitochondrial genome could lead to functional effects on the cellular level. Only a mosaic distribution of mutated genomes, resulting from preferential accumulation of mutants in certain cells, can explain the occurrence of such functional effects in these cells. To cause a functional effect within a cell, a pathogenic point mutation must typically exceed 85–90% heteroplasmy, while deletions appear to cause functional effects at heteroplasmy levels above only 60% [16]. This threshold concept has been validated in tissue samples from numerous patients with mitochondrial diseases harboring pathogenic point mutations or mtDNA deletions, which contain a mosaic of cells with defects in oxidative phosphorylation (OxPhos) that are usually detectable by testing for missing cytochrome *c* oxidase (COX). Similar mosaics of cells that do not have COX have been reported in postmitotic tissues, such as skeletal muscle [17,18], heart muscle [19], or the brain [9,10] However, the number of cells lacking COX in these cases is much lower than that reported in cases of mitochondrial diseases. First attempts have been made to clarify the potential physiological impact of low amounts of cells lacking COX on intact tissues. In research studying such effects on mouse hearts, compelling evidence has been provided that if the frequency of deletions in a small number of individual heart cells exceeds the abovementioned threshold, then arrhythmia [20]—a typical symptom of age-related heart disease—may develop. Similarly, it is easy to imagine that individual neurons with impairment of OxPhos, which have been detected in many central nervous system (CNS) disorders and in the aging brain [9,10], can affect the function of complex neuronal networks. However, this hypothesis [21] remains to be investigated and further substantiated.

In light of the above challenges of the mitochondrial theory of aging, we would like to critically address the issue of the role of oxidative stress in mtDNA-dependent aging in the present review.

## 2. Sources of Reactive Oxygen Species: Mitochondria versus NAD(P)H Oxidase

The mitochondrial respiratory chain is a well-known source of ROS [22]. Respiratory chain Complexes I (its flavin (FMN) moiety, [23]) and III (the complex-associated semiquinone radical, [24]) are able to transfer an electron from one of their redox centers to molecular oxygen resulting in superoxide production. The formed membrane impermeable superoxide anion is rapidly converted by superoxide dismutases (SOD2 in the matrix and SOD1 in the intermembrane space) to H_2_O_2_. Direct quantitative in vitro measurements with Amplex red-based assays show that isolated rat brain mitochondria can generate (mainly via energy-dependent reverse electron flow reaction) at a rate of not more than 800 pmol H_2_O_2_/min/mg mitochondrial protein, which corresponds to approximately 1.6% of the maximal oxygen consumption [23]. It remains doubtful, however, if the mitochondrial superoxide generation, which approaches the high levels mentioned above only under conditions of energy-dependent reverse electron flow, is the only relevant source of ROS, because it is known that under direct-electron-flow conditions the mitochondrial rate drops to about 40 pmol H_2_O_2_/min/mg of mitochondrial protein [23]. One has also to consider that the NAD(P)H-oxidase of neuronal debris-activated microglial cells can generate up to 45 pmol H_2_O_2_/min/mg of cellular protein in phagocytosis-associated oxidative bursts [25]. This value is very similar to the maximal ROS production measured in isolated rat brain mitochondria under conditions of energy-dependent reverse electron flow, taking into consideration that microglial cells contain about 0.05 mg mitochondrial protein. Clearly, this microglia-generated extramitochondrial H_2_O_2_ could also potentially contribute to mitochondrial ROS-induced damage in neighboring neurons. This is consistent with the observation that intact tissues, such as the perfused liver (a tissue rich in peroxisomes), can generate between 50 and 490 nmol H_2_O_2_/min/g wet weight [22]. Because 1 g liver contains only about 50 mg mitochondrial protein, this high rate cannot be explained by assuming ROS production only by mitochondria. However, the amount of the extramitochondrial contribution to overall ROS production is obviously tissue-dependent and might be substantially lower in mitochondria-rich cells, like cardiomyocytes. Moreover, even other mitochondrial ROS generators that are not constituents of the respiratory chain, such as monoaminooxidase, α-glycerophosphate dehydrogenase, or α-ketoglutarate dehydrogenase, might substantially contribute to the overall ROS production in a tissue-specific manner [26,27].

H_2_O_2_ is membrane permeable via aquaporin channels [28]; however, similar to poor membrane permeable superoxide, hydrogen peroxide is not able to produce significant oxidative damage to biomolecules in the absence of free Fe^2+^. In the presence of this cation, H_2_O_2_ is reduced to the highly reactive HO·radical by Fenton chemistry:

Fe^2+^ + H_2_O_2_ → Fe^3+^ + HO^•^ + OH^−^

It is well known that the mitochondrion is an important hub for cellular iron metabolism and that transport of iron into the mitochondria is directly coupled to uptake of the metal at the cell membrane [29]. This is due to the fact that the biosynthesis of iron-sulfur clusters and heme groups is localized in the mitochondrial matrix space. Even though most free iron ions in the mitochondrial matrix are bound to frataxin and a mitochondrial ferritin, which is similar to the cytosolic H-ferritin [30], low concentrations of free iron in the matrix can initiate a Fenton reaction. The presence of free iron ions (chelatable iron) was shown in mitochondria (12–16 µM) [31,32] and cytosol (6 µM) [33] of rat hepatocytes. Interestingly, the brain area with the highest detectable level of mtDNA mutagenesis—the substantia nigra from aged individuals [10] and from individuals with Parkinson’s disease (PD) [9,34]—appears to contain the highest levels of iron [35]. Therefore, apart from the vicinity of superoxide generating sites in respiratory chain, the distribution of iron also supports a theory of ROS-induced preferential damage of biomolecules, such as DNA, in the mitochondrial compartment.

## 3. Effects of ROS on Mitochondrial DNA

**Figure 1:**
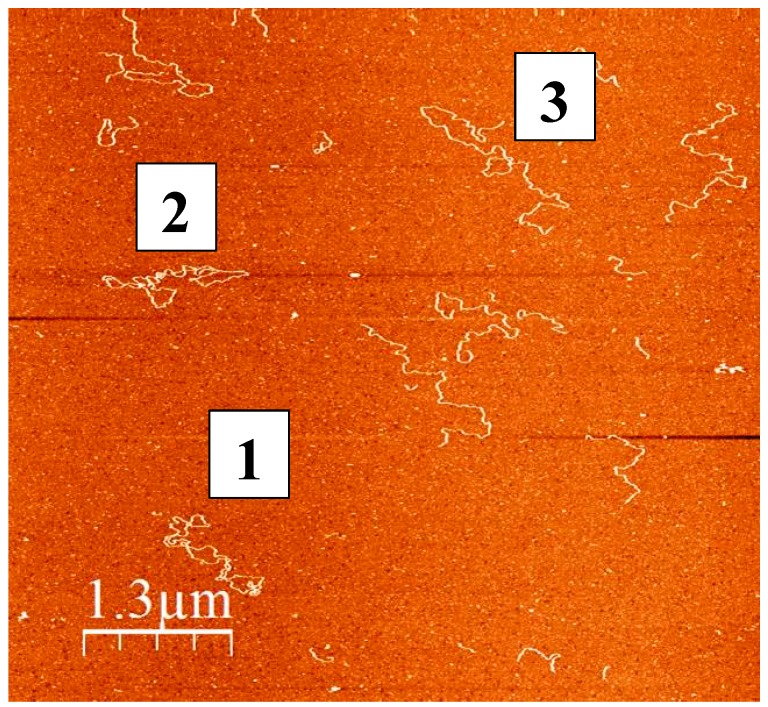
Atomic force microscopy (AFM) image of mitochondrial DNA (mtDNA) isolated from human skeletal muscle (essentially as described by [36]) by phenol extraction. The DNA molecules (0.6 ng/mL) were deposited on freshly cleaved mica in 4 mM HEPES-K (pH 7.4), 2 mM MgCl_2_, and 10 mM NaCl for 5 min. The surface was rinsed with ultrapure distilled water and dried by blowing nitrogen gas. AFM imaging was performed on a Solver PRO AFM system (NT-MDT, Moscow, Russia), in a semicontact (tapping) mode, using Si-gold-coated cantilevers (NT-MDT) with a resonance frequency of 80–110 kHz. Molecules 1 and 2 are supercoiled and molecule 3 is linear mtDNA, all having a contour length of 5 µm.

Preparations of human mtDNA are known to contain a large fraction of supercoiled DNA, while linearized and nicked forms of the nucleic acid are considered to be a result of the damage caused by ROS, other intracellular effectors, or the isolation procedure. The complexity of mtDNA molecules isolated from human skeletal mitochondria can be visualized by atomic force microscopy (see Figure 1). Molecules 1 and 2 represent the classical supercoiled mtDNA having a contour length of 5 µm (which corresponds to about 16 kb canonical double stranded DNA), while molecule 3 is linear mtDNA of the same length. The smaller DNA molecules are either mtDNA fragments occurring as result of isolation-related damage or contaminating nuclear DNA fragments.

**Figure 2:**
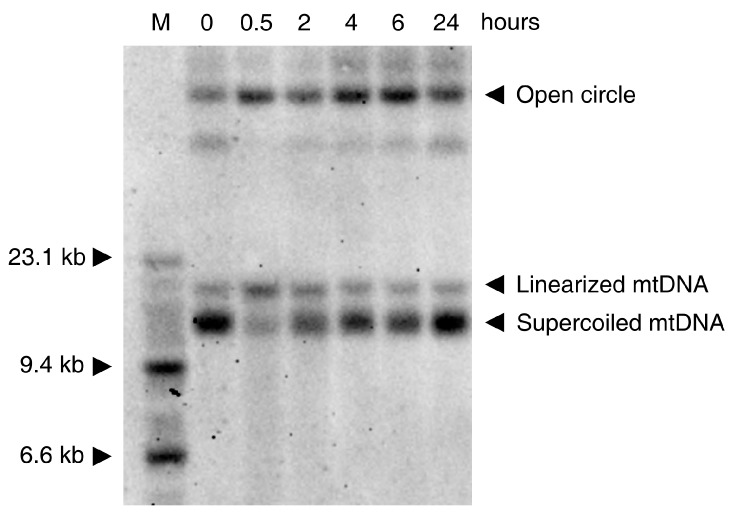
Southern blot showing the time course of native mtDNA damage caused by 1 mM H_2_O_2_ and followed by the DNA repair by HEK293 cells. M: molecular weight marker; 0: control DNA (from untreated cells); The cells were exposed to 1 mM H_2_O_2_ and harvested after 30 min, 2 h, 4 h, 6 h, 24 h, respectively. 1 µg of total DNA was loaded on a 0.6% agarose gel prepared in tris-borate-EDTA (TBE) buffer and was run in the presence of 0.5 µg/mL ethidium bromide overnight at 40 V. After alkaline treatment and re-neutralization of the gel, the DNA was blotted to a Zeta-Probe membrane (Bio-Rad, Hercules, CA, USA) and immobilized by baking at 80 °C for 30 min. The blot was hybridized with a MT-ND6 probe.

In vitro experiments show that the oxidative damage of mtDNA is seen in various cell culture models only at rather high concentrations of ROS (depending on cell type and medium composition). This low sensitivity to ROS is probably related to the coating of mtDNA with Tfam (mtTFA)—the mitochondrial histone [37]. While H_2_O_2_ and the superoxide anion cause no direct damage to the DNA [38], the HO· radical formed from H_2_O_2_ by the Fenton reaction (see above) shows strong mutagenicity [39] through the formation of thymine glycol (from pyrimidine bases) and 7,8-dihydro-8-oxo-2′-deoxyguanosine (8-oxodG, from guanine) [40,41]. An additional mutagenic effect of the hydroxyl radical is the formation of single-strand breaks and more rarely, double-strand breaks, affecting the entire integrity of mtDNA. Usually H_2_O_2_ concentrations above 1 mM are required to obtain clearly detectable effects. A typical Southern blot experiment showing the effects of H_2_O_2_ on HEK293 cells is presented in Figure 2. The effect on the DNA is clearly visible at the very early stage of the process. Thirty min treatment of the cells with 1mM H_2_O_2_ leads to disappearance of the supercoiled mtDNA and appearance of open circles and linearized mtDNA molecules (Figure 2).

This result is in accordance with previous pioneering work [39], showing that the primary effect of H_2_O_2_ is due to damaging of mtDNA by a hydroxyl radical formed by a Fenton reaction. In addition to the earlier-reported creation of nicks in the strands of the DNA circle, a considerable amount of linearized mtDNA is present shortly (30 min) after exposure of the cells to H_2_O_2_ (Figure 2). At later time-points, the recovery of circular mtDNA at the expense of linear and nicked mtDNA species is visible. This relatively fast recovery can be explained by the repair of the DNA (re-ligation of linearized mtDNA molecules and repair of single-strand breaks by base excision repair) as well as by de novo DNA synthesis, because the added hydrogen peroxide is rapidly degraded under cell culture conditions [42].

## 4. Somatic Mitochondrial DNA Mutations in Aging: Free Radical Related Mutagenesis versus POLG Errors?

The well-documented ROS-induced modification of the DNA bases has been considered to be one of the main reasons for ROS-induced mutagenicity. 7,8-Dihydro-8-oxo-2′-deoxyguanosine (8-oxodG) formed as a result of ROS, triggers the mispairing of 8-oxodG with adenine, leading to G > T transversion mutations [43]. Thymine glycol, which is also formed by DNA exposure to oxidizing agents or radiation-generated free-radicals, is not mutagenic but causes a strong replication block [44]. However, much evidence has been obtained that the somatic mitochondrial point mutation spectrum of aged individuals is characterized by a strong G > A transition mutation preference and that the DNA contains only a very low percentage of G > T transversion mutations [45,46]. This is not only valid for humans but has also been shown for *Drosophila melanogaster* [13]. This is apparently not in line with the mitochondrial theory of aging, because the amount of ROS-related 8-oxodG associated mutations in mtDNA is low and age-independent [3]. However, this apparent contradiction can be solved by taking into account the fact that the specific nucleotide selectivity of POLG sufficiently prevents the fixation of G > T transversion mutations even after abundant formation of 8-oxodG [47]. According to this work, the incorporated 8-oxodG causes an approximately 95% replication blockade, but the remaining insertion of nucleotides occurs in the order dCTP >>> dATP > dGTP > dTTP, such as in the case of unmodified dG. Due to this fact, the selectivity of POLG allows it to cope with high 8-oxodG levels in the mitochondrial genome making the 8-oxodG positions less mutagenic. Therefore, the low abundance of somatic G > T transversion mutations in the mtDNA of aged individuals or individuals with Alzheimer’s disease (AD) as observed in [45,46] cannot be used as an argument against potentially relevant oxidative mtDNA damage in the aging process [45]. Because the contribution of 8-oxodG to oxidative damage-related mutagenicity is low, other, less abundant base modifications, such as nucleotide deamination, still might contribute to the age-related point mutation spectrum of mtDNA.

As an alternative explanation for the increased somatic mtDNA point mutation load in the aging process, the limitations in fidelity of POLG have been widely discussed [2,3,48]. This seems, at least qualitatively, to correlate with in vitro data that the nucleotide discrimination factor for the misinsertion of dT opposite to dG (leading to a G > A transition mutation) is fivefold lower than for misinsertion of dA (causing a G > T transversion mutation) [47]. However, according to this work, an approximately 30-fold difference of the nucleotide discrimination factor would be required to explain the high frequency of G > A transition mutations in relation to the low abundant G > T transversion mutations detected in tissue samples [45,49]. Other data for human POLG fidelity [50] indicate a closer match to the measured nucleotide-specific point mutation frequencies.

The following experimental evidence suggests that mtDNA point mutation loads are associated with the aging process: (i) increased levels of somatic point mutations in aged individuals [5,6] (the observed spectrum of somatic point mutations [45,46] roughly corresponds to the spectrum of point mutations detected in the human phylogeny with a high prevalence of G:C > A:T transition mutations); (ii) lack of the proofreading activity of POLG that results in high loads of somatic point mutations in mtDNA, causes accelerated aging in mice [14,15]; and (iii) apart from the findings on humans and mice, mtDNA mutations also appear to be relevant to replicative senescence in yeast, because budding yeasts lose their functional mtDNA late in life [51]. 

For mice, accelerated aging appears to be exclusively related to the point mutation load, because mice with wild type POLG, which inherited only the increased mtDNA point mutation load, also have a considerably reduced life span [52,53]. However, even though these mice have a phenotype reminiscent of accelerated aging, there are the following differences to observations in aged human individuals: (i) The point mutation load in these mice (about tenfold higher than in age-matched control mice) is one order of magnitude higher than that observed in tissue samples from aged individuals [54]; (ii) Heterozygous p.D257A POLG mice, which also have considerably elevated point mutation loads (fivefold higher than in the control animals), show no premature aging phenotype [14]; and (iii) The distribution pattern of point mutations appears to be different with respect to aged human subjects. While aged human tissue samples show approximately 10-fold higher mutation loads in the D-loop region, in mutator mouse tissues, higher point mutation loads have been detected in the coding region [14]. Moreover, mutator mouse tissues show substantially elevated levels of A:T > T:A transversions compared to aged subjects [55]. This corresponds to the alterations of the somatic mtDNA point mutation spectrum observed in yeast mutants lacking the mtDNA polymerase γ proofreading activity, which show increased levels of transversion mutations, in particular of A:T > T:A type [56,57]. The contribution of the increased point mutation load in the mutator mouse to ROS production is discussed controversially. In vitro experiments conducted on mutator mouse tissues showed no increased ROS production [58] or signs of oxidative damage [14,15] apparently contradicting the free radical theory of aging [59,60]. On the other hand, overexpression of mitochondrial catalase [61], treatment with *N*-acetyl cysteine [62], endurance exercise [63], or treatment with the mitochondrially targeted antioxidant SkQ1 [64] delayed the onset of aging in this mouse model. This discrepancy can be explained assuming a very moderate effect of point mutation load on mitochondrial ROS production. The level of ROS reached in the mitochondria might be not high enough to cause damage; however, it is sufficient to affect pro-apoptotic and pro-inflammatory redox signaling pathways [65].

## 5. Somatic Mitochondrial DNA Deletions

One of the key observations promoting the idea that mitochondrial dysfunction plays a role in aging was the increased frequency of respiratory chain-deficient cells in the aged human heart [19]. This phenomenon was also confirmed in other postmitotic tissues, such as skeletal muscle and the brain [17,66]. In colon crypts [67] and liver [68], COX-negative regions representing a single stem cell-derived cluster of cells have been described in aged individuals. Analysis of the mtDNA in single COX-negative cells revealed mainly high amounts of single or multiple mtDNA deletions and, less frequently, potentially pathogenic point mutations [69,70,71]. This suggests that accumulation of mtDNA deletions is at least as relevant in aging-related mitochondrial dysfunction as point mutations.

In aging individuals, a broad spectrum of multiple deletions (a few percent of the total mtDNA) are present in postmitotic tissues. Some of these deletions, distributed between cells in a highly heterogenic manner, cause respiratory deficiency in a small fraction of cells. It has been demonstrated on mouse hearts that, in a complex functional network of cells, even a small number of dysfunctional units can lead to arrhythmia and, as result, to heart failure [20]. In these experiments mutant Twinkle helicase was used to generate aged-dependent accumulation of deletions in the mtDNA of the animals and to mimic the situation in aged humans.

It is a generally accepted observation that in patients carrying single mtDNA deletions, deletion breakpoints often are flanked by direct-sequence repeats. Replication slippage has been hypothesized to be responsible for this phenomenon [72]. Another pathway that may play an essential role in generating deletions in mtDNA is associated with the repair of double-strand breaks in the damaged mtDNA [73]. The first and, up to now, only experimental evidence that mtDNA damage can create deletions was obtained in a mouse model in which double-strand breaks were introduced by a mitochondrial-targeted endonuclease, mitoPstI [74]. Linearization of mtDNA led to the generation of deletions with one breakpoint located close to the original cleavage site and the other in the vicinity of the D-loop. Interestingly, similar combinations of the breakpoints have been described in the sclerotic hippocampus [49,75], leading to the hypothesis that mtDNA double-strand breaks, possibly generated by oxidative damage, play a role in this pathological process. To find the common denominator between double-strand break repair and the presence of repeat-associated breakpoints, it has been suggested that homologous recombination or microhomology-mediated end joining is the underlying mechanism [73,76]. However, in several cases the majority of breakpoints are not associated with direct repeats, as observed in different tissues of the mitoPstI mouse [74,77] or deficiencies of the mitochondrial replication machinery [78,79]. This might point to the existence of alternative pathways of deletion generation, such as ligase III-mediated non-homologous end-joining of the free ends of linear mtDNA molecules.

As noted above (paragraph 3), the ROS attack on mtDNA leads preferentially to the formation of single-strand breaks, increasing the fraction of nicked circular mtDNA molecules (Figure 2). However, a substantial increase in the amount of linear mtDNA molecules can also be observed 30 min after treatment with H_2_O_2_ (Figure 2). Linearized mtDNA molecules can be either degraded [77,80] or re-ligated. The latter reaction, if occurring after partial resection of the free DNA ends, can yield deleted mtDNA molecules (detailed mechanisms of deletion formation after double-strand breaks are discussed in [73]). Thus, ROS-induced formation of mtDNA double-strand breaks seems to be a suitable mechanism for the generation of somatic mtDNA deletions. Because deletions are abundant in iron rich areas of the brain, such as the substantia nigra [9,10], and preferably detected under conditions of elevated oxidative stress, such as in inflammatory CNS disorders [49,81], ROS-induced mtDNA deletion formation followed by their segregation-dependent accumulation appears to be a valid mechanism to explain the age-dependent decline of oxidative capacity in individual cells.

The most intensively investigated mouse model of mtDNA-related aging is the mutator mouse, in which a mutation of the replicative DNA polymerase POLG diminishes the exonuclease activity of the enzyme [14,15]. As discussed earlier, the effect of the acceleration of somatic point mutation accumulation in mtDNA has been extensively studied in this model. Whether mtDNA deletions also influence the phenotype of the mutator mouse is still not sufficiently addressed in the literature. Importantly, the presence of linear and rearranged mtDNA species have been demonstrated in the mutator mouse [60,82]. While in humans the presence of individual respiratory deficient cells is a hallmark of mtDNA-related mitochondrial dysfunction in disease and aging, aged or mutator mice display very few respiratory deficient fibers in their skeletal muscle. This makes it difficult to guess about the relative contribution of point mutations to the phenotype in comparison to other types of mtDNA alterations.

## 6. Somatic Mitochondrial DNA Mutations in Neurodegenerative Diseases

In PD mitochondrial abnormalities in brain samples, including a severe Complex I deficiency, have been reported by many groups [83]. In particular, in substantia nigra neurons of PD patients, elevated levels of mtDNA deletions have been detected [9,84]. Similarly, elevated deletion loads in this iron-rich brain area of aged individuals [10] were reported. Importantly, the level of heteroplasmy of individual mtDNA deletions observed in single neurons was above the threshold level (see paragraph 1), which is sufficient to explain the lack of cytochrome *c* oxidase activity staining [10]. It was shown recently [84] that in addition to higher deletion levels in individual substantia nigra neurons of PD patients compared to healthy individuals, the wild type mtDNA copy number is also decreased, resulting in a gradual takeover by deleted mtDNA molecules. In apparent contrast to earlier work [10], recent ultradeep sequencing analysis of single substantia nigra neurons showed that they contain, in addition to a single highly abundant deleted mtDNA species, a complex spectrum of deletion species [85]. Another study [34] reported elevated somatic point mutation loads in the substantia nigra neurons of early PD patients with about a threefold increase of G > T/C > A transversions, while in late PD patients the somatic point mutation spectrum was apparently not different to controls. Based on these results, in support of the assertion that ROS is a potential mutagenic factor, it was suggested that neurons with high mutation levels degenerate and thus are absent in late stage PD tissues [34].

In AD, the most common late-onset neurodegenerative disorder, mitochondrial function is negatively affected by Aβ fragments, suggesting that the mitochondrial dysfunction is a consequence of Aβ toxicity [86,87,88]. Additionally, for AD, several groups have reported increased point mutation loads of mtDNA, particularly in the hippocampal formation [46,89]. Again, the point mutation spectrum is comparable to that detected in normal aging with a high prevalence of transition mutations [46]. The low content of G > T transversions has been interpreted to be inconsistent with a canonical mutation signature of oxidative damage to mtDNA and, thus, nucleotide deamination or POLG-associated replication errors have been suggested to be a potential reason for these somatic mutations [46,90]. However, as previously noted, the low level of G > T transversions might be a consequence of the specific nucleotide selectivity of POLG [47,91] and do not necessarily implicate a low 8-oxodG content in AD brain tissue samples.

Mitochondrial abnormalities have been also reported for conditions of selective neurodegeneration, such as in the case of temporal lobe epilepsy with Ammons horn sclerosis (AHS) [92], where a selective death of pyramidal neurons in the hippocampal formation takes place. Here, the molecular basis for the mitochondrial dysfunction is related to both mtDNA depletion [93] and accumulation of mtDNA deletions [49]. Interestingly, the accumulation of deletions in AHS appears to be closely related to the presence of immune cells, pointing to oxidative stress as a potential cause for deletion formation under these particular circumstances. The putative mutagenic effect of ROS is visible in the somatic point mutation spectrum of the hippocampus of AHS patients, in which at least a slight elevation of G > T transversions has been observed [49].

Similarly to AHS, in multiple sclerosis (MS)—a chronic, inflammatory disease caused by the loss of myelin and gliosis—mitochondrial abnormalities have been detected. In patient brains, a reduction of activities of Complexes I and III, as well as a decrease in COX staining of neurons compared to age-matched controls has been described [94,95]. In the choroid plexus of MS patients, substantially elevated levels of mtDNA deletions have been observed [81,96]. Here, similarly to AHS, the somatic mutations of mtDNA seem to be closely linked to ROS generated by immune and microglial cells.

## 7. Conclusions and Future Directions

Although there is compelling evidence that the somatic point mutation spectrum of aging tissues lacks G > T transversions, it cannot be used as an argument for the missing contribution of ROS to aging because this is likely related to the nucleotide selectivity of POLG. In contrast to point mutations, the age-dependent accumulation of mtDNA deletions is still compatible with the direct mutagenic effects of ROS. Linearized mtDNA, the prerequisite for deletion formation, can be generated by the attack of HO· radicals formed from H_2_O_2_ by a Fenton reaction. The source of mtDNA-damaging ROS remains less clear because the mitochondrial contribution to tissue ROS production seems to be lower than previously thought. Importantly, the ROS generation by inflammation-linked processes and the distribution of free iron also require careful consideration and future investigations.
